# Application of Pharmacokinetics Modelling to Predict Human Exposure of a Cationic Liposomal Subunit Antigen Vaccine System

**DOI:** 10.3390/pharmaceutics9040057

**Published:** 2017-12-07

**Authors:** Raj K. S. Badhan, Swapnil Khadke, Yvonne Perrie

**Affiliations:** 1 Aston Pharmacy School, School of Life and Health Sciences, Aston University, Birmingham B4 7ET, UK; r.k.s.badhan@aston.ac.uk; 2 Strathclyde Institute of Pharmacy and Biomedical Sciences, University of Strathclyde, Glasgow G4 0RE, UK; swapnil.khadke@strath.ac.uk

**Keywords:** pharmacokinetics, physiologically based pharmacokinetics, liposome, antigen, adjuvant, vaccine

## Abstract

The pharmacokinetics of a liposomal subunit antigen vaccine system composed of the cationic lipid dimethyldioctadecylammonium bromide (DDA) and the immunostimulatory agent trehalose 6,6-dibehenate (TDB) (8:1 molar ratio) combined with the Ag85B-ESAT-6 (H1) antigen were modelled using mouse in-vivo data. Compartment modelling and physiologically based pharmacokinetics (PBPK) were used to predict the administration site (muscle) and target site (lymph) temporal concentration profiles and factors governing these. Initial estimates using compartmental modelling established that quadriceps pharmacokinetics for the liposome demonstrated a long half-life (22.6 days) compared to the associated antigen (2.62 days). A mouse minimal-PBPK model was developed and successfully predicted quadriceps liposome and antigen pharmacokinetics. Predictions for the popliteal lymph node (PLN) aligned well at earlier time-points. A local sensitivity analysis highlighted that the predicted AUC_muscle_ was sensitive to the antigen degradation constant k_deg_ (resulting in a 3-log change) more so than the fraction escaping the quadriceps (f_e_) (resulting in a 10-fold change), and the predicted AUC_PLN_ was highly sensitive to f_e_. A global sensitivity analysis of the antigen in the muscle demonstrated that model predictions were within the 50th percentile for predictions and showed acceptable fits. To further translate in-vitro data previously generated by our group, the mouse minimal-PBPK model was extrapolated to humans and predictions made for antigen pharmacokinetics in muscle and PLN. Global analysis demonstrated that both k_deg_ and f_e_ had a minimal impact on the resulting simulations in the muscle but a greater impact in the PLN. In summary, this study has predicted the in-vivo fate of DDA:TDB:H1 in humans and demonstrated the roles that formulation degradation and fraction escaping the depot site can play upon the overall depot effect within the site of administration.

## 1. Introduction

The liposomal system composed of the cationic lipid dimethyldioctadecylammonium (DDA) and the immunomodulating glycolipid trehalose dibehenate (TDB) is a two-component adjuvant system known as CAF01. The CAF01 system has been shown to be effective in producing protective immune responses against pathogens such as chlamydia, malaria, influenza and tuberculosis (TB) [1,2]. Korsholm et al. (2007) described that one of the key mechanisms behind this immunomodulatory effect results from the cationic charge of the vesicles, which electrostatically binds and enhances antigen uptake by antigen-presenting cells via actin-dependent endocytosis [3]. This action works in synergy with the immunostimulation provided by TDB; TDB is a synthetic analog of trehalose 6-6-dimycolate, an immunostimulatory component of *Mycobacterium tuberculosis*, a mincle-recognised ligand and its adjuvant effect in vivo is MyD88 dependent [4].

When considering the biodistribution of these cationic liposomal adjuvants after immunisation, research from our group has shown that DDA:TDB liposomes are retained at the injection site and that these vesicles promote the retention of antigen at the site of injection, thus promoting co-delivery of both liposomal adjuvant and antigen to appropriate antigen-presenting cells [5]. A stable and reproducible dual-radiolabelling method (whereby the adjuvant (liposome) is labelled with ^3^H and the antigen (a subunit protein) with ^125^I) was used to track liposomes and antigen in vivo [6]. The depot effect was found to be dependent on the cationic nature of vesicles; when DDA was replaced with neutral lipid disteoryl phosphatidylcholine (DSPC), no depot was formed at site of injection and lower levels of immune responses were noted [7]. It has also been shown that DDA:TDB vesicles from ~200 to 1500 nm have similar clearance kinetics from the injection site. This suggests that with these cationic systems, size reduction does not modify clearance kinetics [8]. This could be a result of aggregation of these cationic liposomes after injection. Furthermore, whilst not modifying the clearance rates of the vesicles, the presence of TDB within the liposomes promotes increased recruitment of monocytes to the depot, again demonstrating the synergistic delivery and stimulatory action of the DDA:TDB formulation [5]. However, clearance rates from the injection site can be increased by PEGylation of these cationic liposomes [9,10]. Due to the hydrophilic chains of the polyethylene glycol (PEG) extending out from the surface of the liposomes, the cationic charge of the DDA is masked and hence aggregation is blocked [9]. This results in a faster drainage of the liposomes from the site of injection compared to non-PEGylated liposomes [9,10].

Despite these recognised links between distribution profiles and vaccine efficacy, the application of pharmacokinetic modelling and simulation to vaccines is sparse. This often results from a lack of understanding of how traditional pharmacokinetic terms developed for low molecular weight agents can be correlated to antigen dose, safety and efficacy, and is supported by a lack of appropriate correlations to efficacy. A complete understanding of the pharmacokinetics of liposomal formulations is limited, and modelling approaches to support formulation development is still in its infancy as a discipline. However, recently, a number of groups have begun to develop mechanisms by which to understand these processes in the context of pharmacokinetic modelling and simulation [11,12,13,14,15,16,17]. Whilst the pharmacokinetics of the Ag85B-ESAT antigen has not been assessed in humans, a liposome formulation with the antigen has undergone phase-1 trials demonstrating safety and tolerability [18,19]. The purpose of this study was to explore the potential to apply the principles of pharmacokinetic modelling and simulation to the analysis of the kinetics of liposome–antigen disposition data generated in mice through traditional compartmental modelling and physiologically based pharmacokinetics (in humans).

## 2. Materials and Methods

### 2.1. Data Collection

Data previously generated by our group from a dual radio-labelled DDA:TDB liposome–adjuvant (Ag85B-ESAT-6) formulation [9,20,21,22] was used for these pharmacokinetics studies. Biodistribution data obtained from these previous studies was used as either relative to the percent dosed or converted to mass units (300 µg liposome and 2 µg antigen) for dosing. During these studies from which the data was used, radio-labelled antigen and liposomes were used as previously outlined [6]. Briefly, the antigen was radio-labelled with ^125^I using Pierce iodination tubes containing Pierce iodination reagent. The tubes contain an oxidizing reagent which converts NaI into a reactive iodine molecule that can insert into the tyrosol group of tyrosine amino acids. Liposomes were radio-labelled using commercially available tritiated lipid dipalmitoyl phosphatidylcholine (DPPC). Addition of this tracer lipid did not affect the physicochemical properties of DDA:TDB. Liposome membrane stability and retention of the radiolabel tracer upon exposure of liposomes to the in-vivo environment was previously confirmed using stability studies at 37 °C in a high-protein environment (50% Fetal Calf Serum (FCS)) [5]. Mice were injected with Ag85BESAT-6 (radiolabeled with ^125^I)-adsorbing liposome (radiolabeled with ^3^H) formulations (50 μL/dose, im (intramuscular) injection). At 1, 4 and 14 days post-injection (pi), mice were terminated by cervical dislocation and tissue from the site of injection (SOI), and local draining lymph nodes (LNs) were removed for analysis of liposome (^3^H) and antigen (^125^I) using methods previously described elsewhere [6].

In the studies where the in-vivo data were used in the modelling, liposomes were composed of DDA:TDB at a weight ratio of 5:1. Mice received 50 μL/dose which contained 250 μg DDA, 50 μg TDB and 2 μg of antigen. Liposomes were in the range of 400 to 600 nm in size (z-average diameter of 481 ± 20 with a PDI of 0.23 ± 0.1) and highly cationic in nature (55 to 65 mV).

### 2.2. Compartmental Modelling of Data

**Figure 1:**
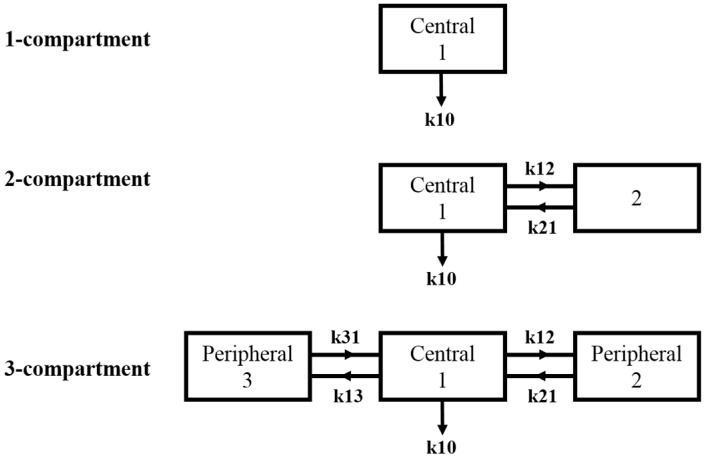
Schematic diagram of compartmental model. Boxes represent non-physiological compartments within which the drug is capable of distributing. Arrows indicate direction of transfer between compartments with ‘k’ indicating a transfer rate constant and the numerals indicating the direction of transport. k10 reflects degradation from a compartment.

A 1-, 2- and 3-compartment model (Figure 1) was used to fit the data for the liposome and antigen to model the kinetics at the site of administration (central compartment). Differential equations described a 1-, 2- and 3-compartment model and were numerically solved using Matlab (The MathWorks Inc., Natick, MA, USA, 2015):

1-compartment model:

(1) $$\frac{\mathrm{dC}1}{\mathrm{dt}}=-k10\cdot C1;$$

2-compartment model:

(2) $$\frac{\mathrm{dC}1}{\mathrm{dt}}=-\left(k10+k12\right)\cdot C1+k21\cdot C2;$$

(3) $$\frac{\mathrm{dC}2}{\mathrm{dt}}=K12\cdot C1-k21\cdot C2;$$

3-compartment model:

(4) $$\frac{\mathrm{dC}1}{\mathrm{dt}}=-\left(k10+k12+k13\right)\cdot C1+k21\cdot C2+k31\cdot C3;$$

(5) $$\frac{\mathrm{dC}2}{\mathrm{dt}}=K12\cdot C1-k21\cdot C2;$$

(6) $$\frac{\mathrm{dC}3}{\mathrm{dt}}=K13\cdot C1-k31\cdot C3;$$

where C_n_: concentration in nth compartment; t: time; k10: elimination rate constant; k12: transfer rate constant from central (1) to peripheral (2) compartment; k21: transfer rate constant from peripheral (2) to central (1) compartment; k13: transfer rate constant from central (1) to peripheral (3) compartment; k31: transfer rate constant from peripheral (3) to central (1) compartment.

Pharmacokinetics parameters were then calculated based on the best-fitting model selected according to the Akaike information criterion (AIC).

### 2.3. Mechanistic Modelling of Data

**Figure 2:**
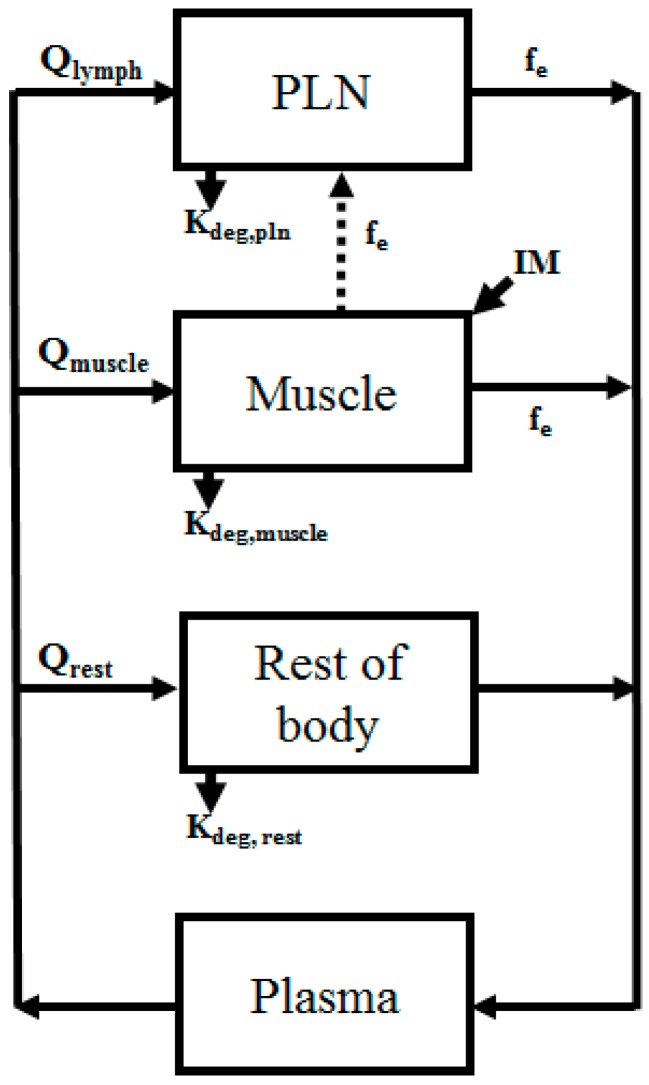
Schematic represent of the semi-mechanistic PBPK model. Arrows indicate flow between organs. Dashed lines indicates lymphatic draining. IM: intramuscular administration.

Minimal-PBPK (physiologically based pharmacokinetics) models of mice (28 g) and humans (71 kg) [23] were developed and accounted for the site of injection (muscles), target site (PLN), rest of body (ROB) and plasma (Figure 2). Compartments were assigned physiological volume parameters and connected through physiological flows (Table 1). A more mechanistic model, with the inclusion of, for example, a tissue partition coefficient, was not developed due to the lack of rich sampling data points to develop/validate the model with.

The following assumptions were made for this model:

- All tissues were modelled based on the total tissue volume (derived from tissue mass), and, where necessary, assuming a density of 1.
- The ‘muscle’ compartment was modeled solely by the quadriceps tissue component in mice and the deltoid tissue in humans.
- The ‘lymph’ compartment was modelled solely by the popliteal lymph node (PLN) with an estimated whole tissue volume of 0.05 mL [28] (assuming a density = 1) in mice. In humans we assumed a mass of 0.05 g/kg body weight, based on a similar relationship in rabbits [27].
- Plasma flow to and drainage from the PLN were assumed to be 0.012% cardiac output [27].
- The fraction escaping (f_e_) the quadriceps and being drained into the PLN was fixed at 3 × 10^−5^ for liposome and 3.6 × 10^−6^ for antigen, to reflect fraction escaping the muscle based upon the average ratio of percent accumulation for all time points in the target tissues compared to the dose administered. The small-pore theory of molecular translocation across a membrane would preclude molecules below 60 nm in size from undergoing transvascular flow across a capillary wall [29,30,31,32].
- Dosing was modelled as a rapid first-order dose into the quadriceps (approximating a bolus dose) with a ka = 10 day^−1^. The human model focused on simulating antigen only and therefore a human dose of 50 µg was modelled.
- In the absence of plasma concentration of both liposome and antigen and the limited muscle and PLN biodistribution data, an attempt to estimate an appropriate tissue partition coefficient was not conducted and transfer of liposome/antigen out of the site of administration was assumed to occur only through exiting via the muscle blood flow (accounting for the fraction escaping) and the transfer via lymphatics. Transvascular flow was therefore modelled as a rate constant (when accounting for tissue volume):
  $${\mathrm{tv}}_{\mathrm{tissue}}=\frac{Q_{\mathrm{tissue}}}{V_{\mathrm{tissue}}}f_{e}.$$

Differential equations describing the drug flow in the model were solved in Matlab (The MathWorks Inc., Natick, MA, USA, 2015) and are detailed as follows:

Plasma:

(7) $$V_{\mathrm{plasma}}\cdot \frac{{\mathrm{dA}}_{\mathrm{plasma}}}{\mathrm{dt}}=-\left(\sum^{\text{​}}Q_{\mathrm{tissue}}\right)\cdot C_{\mathrm{plasma}}+\left(Q_{\mathrm{rest}}\cdot C_{\mathrm{rest}}\right)+\left({\mathrm{tv}}_{\mathrm{muscle}}\cdot C_{\mathrm{muscle}}\right)+\left({\mathrm{tv}}_{\mathrm{lymph}}\cdot C_{\mathrm{PLN}}\right).$$

ROB:

(8) $$V_{\mathrm{rest}}\cdot \frac{{\mathrm{dA}}_{\mathrm{rest}}}{\mathrm{dt}}=-\left(\left(Q_{\mathrm{rest}}\cdot C_{\mathrm{rest}}\right)+\left(k_{\deg,\mathrm{rest}}\cdot C_{\mathrm{rest}}\right)\right)+\left(Q_{\mathrm{rest}}\cdot C_{\mathrm{plasma}}\right).$$

Quadriceps:

(9) $$V_{\mathrm{muscle}}\cdot \frac{{\mathrm{dA}}_{\mathrm{muscle}}}{\mathrm{dt}}=\mathrm{ka}\cdot \mathrm{Dose}-\left(\left({\mathrm{tv}}_{\mathrm{muscle}}\cdot C_{\mathrm{muscle}}\right)+\left(k_{\deg,\mathrm{muscle}}\cdot C_{\mathrm{muscle}}\right)+\;\left({\mathrm{tv}}_{\mathrm{lymph}}\cdot C_{\mathrm{muscle}}\right)\right)+\left(Q_{\mathrm{muscle}}\cdot C_{\mathrm{plasma}}\right).$$

PLN:

(10) $$V_{\mathrm{PLN}}\cdot \frac{{\mathrm{dA}}_{\mathrm{PLN}}}{\mathrm{dt}}=-\left(\left({\mathrm{tv}}_{\mathrm{lymph}}\cdot C_{\mathrm{PLN}}\right)+\left(k_{\deg,\mathrm{PLN}}\cdot C_{\mathrm{PLN}}\right)\right)+\left(Q_{\mathrm{PLN}}\cdot C_{\mathrm{plasma}}\right)+\;\left({\mathrm{tv}}_{\mathrm{lymph}}\cdot C_{\mathrm{muscle}}\right).$$

Given the lack of robust plasma and whole-body temporal concentration data, parameter optimisation was initially avoided as far as possible, except for the optimisation of the degradation rate constant of the formulation from each tissue (k_deg,tissue_). Optimisation was conducted using a non-linear least-squares fitting algorithm based on previously reported data. Final model simulations were confirmed through visual inspection and observed versus predicted plots (for mouse model only). The percent predictive error (PE) was calculated (see Equation (11)) for mouse data only, where C_pred_ is the model-predicted concentration and C_obs_ the observed concentration:

(11) $$\%\;\mathrm{PE}=\frac{C_{\mathrm{pred}}-C_{\mathrm{obs}}}{C_{\mathrm{obs}}}\times 100\%.$$

### 2.4. Parameter Sensitivity Analysis

An uncertainty analysis was performed to determine how variations in model parameters would influence the depot effect in the target site (muscle) for the mouse model only. A local analysis was performed on Q_muscle_, k_deg_ and f_e_, and was scanned over a defined range (Q_muscle_: ±3-log; k_deg_: ±3-log and f_e_: ±4-log). 3D surface plots of the relationship between two input parameters and the AUC_muscle_ were compared in the muscle and the PLN.

A global analysis was also conducted on muscle kinetics for the liposome and antigen, with scanning limits set as detailed above. Monte Carlo simulations with 1000 simulations were performed with the Latin hypercube sampling algorithm for both the mouse and human models. The resultant 50th and 95th percentiles were graphically assessed.

## 3. Results

### 3.1. Compartment Modelling

**Figure 3:**
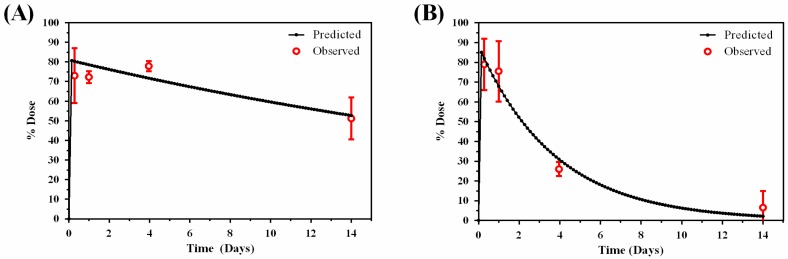
Compartmental modelling estimates for: (**A**) liposome and (**B**) antigen at the muscle.

**Figure 4:**
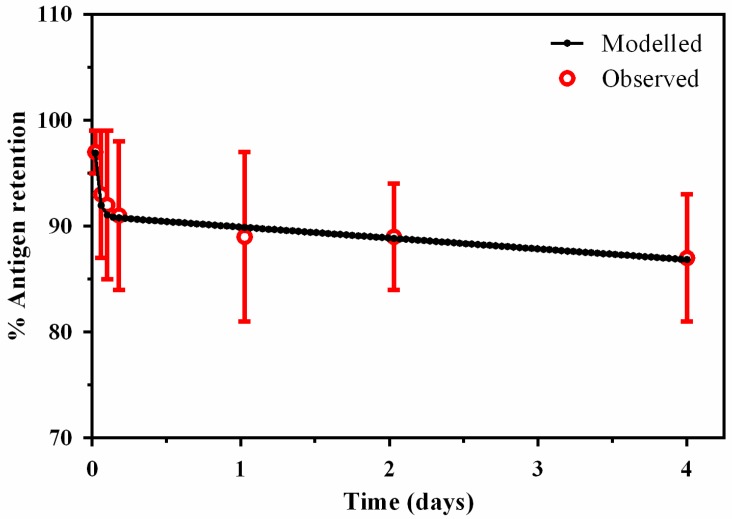
A bi-exponential model of in-vitro antigen stability.

The pharmacokinetics of the liposome formulation was best described by a 1-compartment model with antigen described by a 2-compartment model (Figure 3). Antigen stability was described by fitting in-vitro stability data to a bi-exponential first-order degradation model (Figure 4), demonstrating a terminal half-life of 2.62 days and relatively slow degradation rate of 0.34 day^−1^ (Table 2).

### 3.2. Minimal-PBPK Models

**Figure 5:**
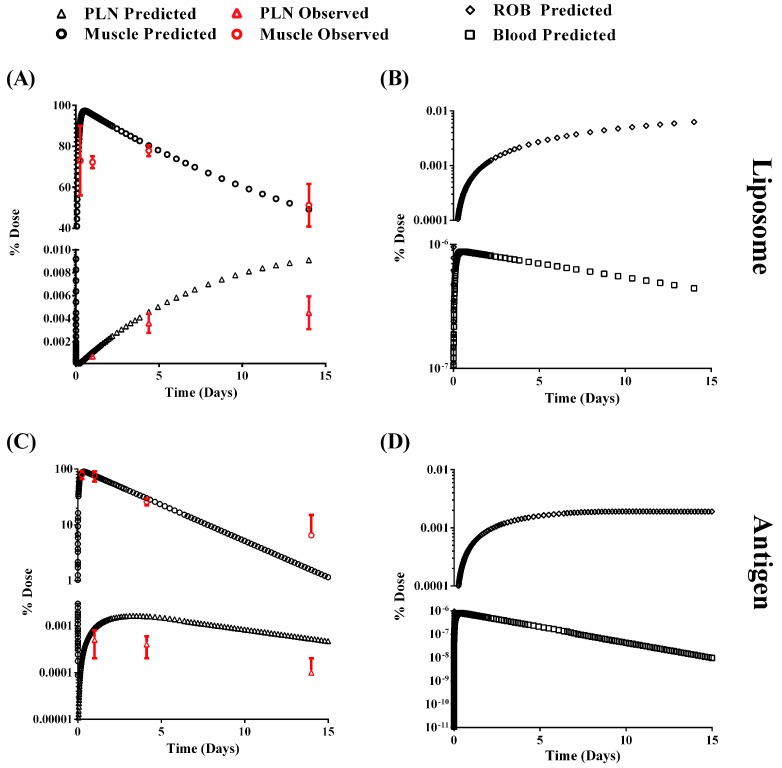
PBPK model predictions for liposome (**A**,**B**) and antigen (**C**,**D**). Muscle and PLN data is shown in (**A**,**C**), and blood and ROB in (**B**,**D**). Red data points represent observed data.

We first aimed at developing a physiological model which best mimicked the kinetics of distributional transfer and degradation kinetics of the formulation at the key target site, namely the muscle. This was further followed on by assessing the ability to predict drainage of the formulation into the local lymph node, that is, the PLN. Modelling was generally deemed to be successful, particularly for the muscle and for both the liposome and antigen. The PBPK model developed was able to capture the kinetics within the compartment with a PE < 33% for the liposome (Figure 5A,B and Table 3). For the antigen, the precision PE was less than 14% for time points 1–4 days, with the final time point showing a larger error (65.9%) (Figure 5C,D and Table 2). In a similar fashion the PLN predictions were also generally successful but demonstrated poorer predictions at 14 days. Fitting of the degradation rate constants yielded estimates that were similar to those from compartment modelling and in-vitro data (Table 4), with antigen demonstrating on average a 10-fold higher rate constant than liposomes.

### 3.3. Sensitivity Analysis

**Figure 6:**
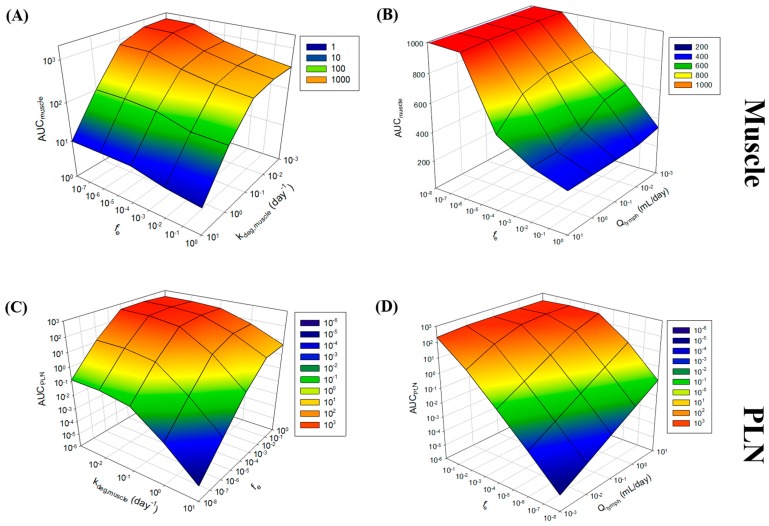
Local sensitivity analysis of f_e_, k_deg_, Q_muscle_ and Q_lymph_ towards AUC_muscle_ (**A**,**B**) or AUC_PLN_ (**C**,**D**). Note the differences in the z-axis scales.

In light of sparse observed data sets, the sensitivity of model input parameters was evaluated through both global and local sensitivity analyses. Local sensitivity analysis was conducted on k_deg_, f_e_, Q_muscle_ and Q_lymph_, where the impact of two of these parameters on AUC_tissue_ was assessed. Analysis was conducted with both liposomes and antigen, with similar trends in sensitivities and therefore liposome trends reported in Figure 6.

The analysis revealed that the AUC_muscle_ was most sensitive to k_deg,muscle_ (Figure 6A), leading to a 2-log magnitude change in AUC_muscle_ over the k_deg,muscle_ range studied (0.001–10 day^−1^), and more particularly beyond 0.1 day^−1^. Our model-fitted estimate for k_deg,muscle_ was at the lower end of this sensitivity. AUC_muscle_ was also highly sensitive to changes in f_e_, with Q_muscle_ not impacting upon the sensitivity of AUC_muscle_ (Figure 6B).

AUC_PLN_ was most sensitive to changes in f_e_, with an almost 8-log-order change in AUC_lymph_ over the range of f_e_ studied (Figure 6C). Q_lymph_ and k_deg,pln_ also resulted in some level of sensitivity on AUC_lymph_ (Figure 6C), however, our estimates for k_deg,pln_ are at the upper limit of the sensitivity of the parameters towards AUC and Q_lymph_ in the mid-range, but in both cases a 50% change in parameter would still provide an estimate of AUC_lymph_ within the same order of magnitude (Figure 6D).

**Figure 7:**
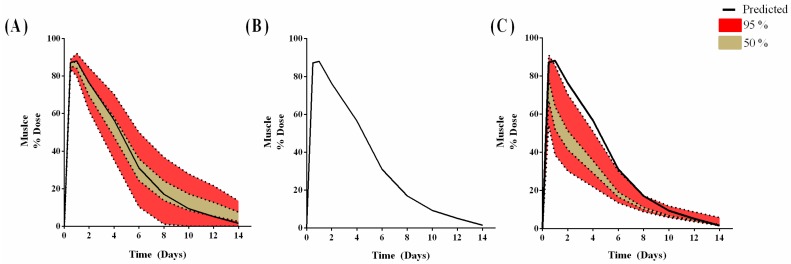
The uncertainty in the antigen muscle concentrations, following 1000 model simulations with a 3-fold change in k_deg,muscle_ (**A**); 1 × 10^−8^ to 1 × 10^−5^ change in f_e_ (**B**) and 1 × 10^−5^ to 1 × 10^−1^ change in f_e_ (**C**) was simulated. The solid line represents PBPK-predicted profiles.

As a result of these local simulations, k_deg,muscle_ and f_e_ were selected for global analysis in the muscle for the antigen (Figure 7). The global analysis revealed a limited uncertainty in the predictions at the earlier time-points for k_deg,muscle_ (Figure 7A), with the uncertainty increasing later in the simulation. With f_e_, sensitivity analysis revealed no impact on predictions with very low f_e_ (<1 × 10^−5^) (Figure 7B), but when f_e_ was increased above 1 × 10^−5^, the uncertainty around the C_max_ increased significantly (Figure 7C) and impacted more on the earlier time points (<6 days).

### 3.4. Human Model

**Figure 8:**
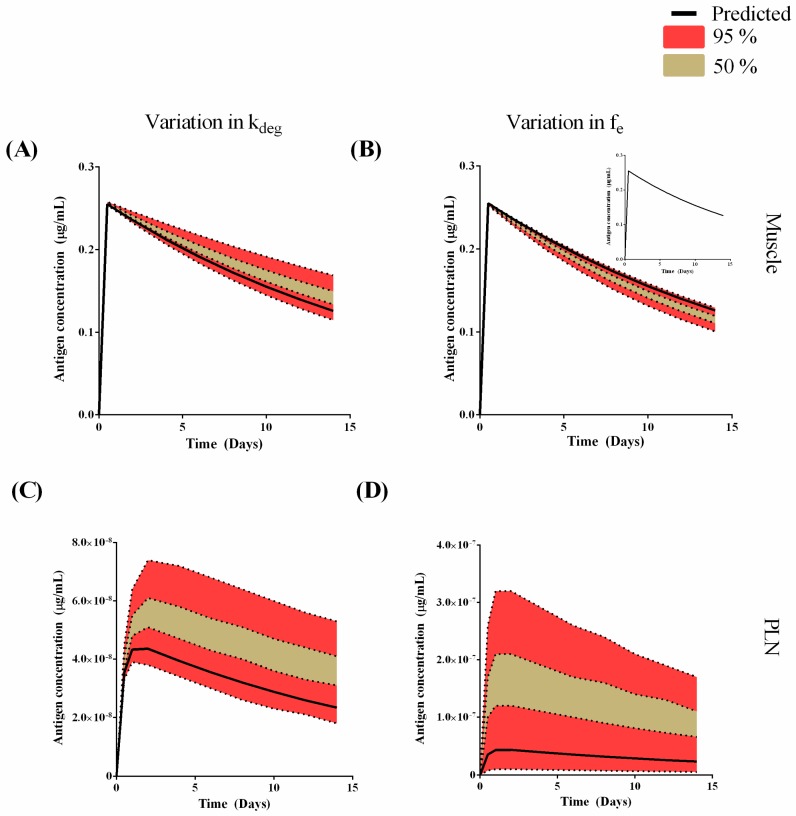
Model predictions (black solid line) and global sensitivity analysis for muscle (**A**,**B**) and PLN (**C**,**D**). (**A**,**C**) show a ± 50% variation in k_deg_; (**B**-insert) and (**D**) show a variation in f_e_ from 1 × 10^−8^–1 × 10^−5^; (**B**) shows a variation in f_e_ from 1 × 10^−5^–1 × 10^−1^.

The mouse model was extrapolated to develop a human PBPK model for the antigen only with the dose adjusted to reflect a single dose administered in adults (i.e., 50 µg). In the muscle, the predicted C_max_ was 0.25 µg/mL with a t_max_ of 0.5 days and half-life of 13.2 days (Figure 8A,B). In the PLN the predicted C_max_ was 0.0438 pg/mL with a t_max_ of 2 days and half-life of 13.2 days (Figure 8C,D). A global sensitivity analysis was then conducted to compare k_deg,tissue_ and f_e_ on muscle and PLN concentration. Uncertainty in the predictions on antigen concertation in the muscle with variation of k_deg_ (±50%) was minimal at the earlier time point, but became more uncertain during the simulations (Figure 8A).

For variations in f_e_, the antigen concentration in the muscle was not sensitive to any change over the range of 1e^−8^ to 1e^−5^ (Figure 8B-insert). However, for the range of 1 × 10^−5^ to 1 × 10^−1^, muscle antigen concentration demonstrated minimal sensitivity and predictions were within the upper 95th percentile (Figure 8B). For the PLN, this uncertainly was significantly greater and predictions were within the lower 95th percentile range for both k_deg_ (Figure 8C) and f_e_ (Figure 8D).

## 4. Discussion

Vaccination plays a key role in the protection of life and promotion of global public health. The use of liposomal vaccine adjuvants offers new approaches to take advantage of the immunomodulatory properties imparted by these systems. This study has focused on demonstrating how mathematical modelling, in the form of pharmacokinetics modelling and simulation, can be applied to gain an early perception of the pharmacokinetic properties of formulation systems for vaccines and adjuvants.

### 4.1. Compartmental Modelling

Compartmental modelling is a widely used empirical tool which can be used to quantify the pharmacokinetics of a molecule of interest from existing data. It makes the assumption that drug distributes instantaneously within a compartment and that the central compartment is often assumed to be the dosing or ‘plasma’ compartment. Compartment modelling in this context models the kinetics within the dosing (quadriceps muscle) compartment in an attempt to describe the pharmacokinetics of both the liposome and the associated antigen.

Compartment modelling demonstrated that the liposome is indeed relatively stable and resides at the site of injection. The antigen on the other hand yielded a 10-fold higher elimination rate (k10) compared to the liposome (Table 2). As k10 is traditionally viewed as an ‘elimination’ process, in this context it can be related to processes driving the reduction of liposome from the dosing compartment. The higher k10 for antigen may suggest either a more-rapid degradation process within the muscle tissue or more-rapid transfer out of the injection site. A significant drawback of compartmental modelling is highlighted in this approach, as it is difficult to assign biochemical or physiological processes to the rate constants that define the compartmental transfer of the formulation. This can include uptake by antigen-presenting cells, which will take up and clear the vaccine from the injection site. Our analysis of the reported in-vitro stability of antigen demonstrates that the antigen was relatively stable in vitro (in the absence of enzymatic degradation processes), with a degradation rate of 0.01 day^−1^. This demonstrates a relatively slow degradation process and leads to a long terminal half-life of approximately 60 days. A key drawback of compartmental modelling is the inability to extrapolate to other species (e.g., humans) and other types of formulations as the very structure of the model is intricately tied up, and its empirical nature requires in-vivo data to model.

### 4.2. Physiological Modelling

Physiologically based pharmacokinetics (PBPK) is an adaptation of compartmental modelling, where the system is described in the context of physiological and biochemical properties, that is, tissue volumes, blood flows and protein/enzymatic expression. We opted to develop a minimal-PBPK model; a model that is semi-physiological and which accounts for the key processes governing formulation disposition at the site of administration and target tissues. Our rationale for choosing this approach, as opposed to a full implementation of a whole-body PBPK model, was driven by: (i) the sparse data available from existing studies for our formulation of choice; (ii) the lack of plasma data which is important when developing a whole-body PBPK model; and (iii) the desire to avoid fitting a large majority of model parameters where the final estimates would undoubtedly be unreliable due to the limited available data.

Our approach developed four compartmental models consisting of blood, rest of body, muscle and popliteal lymph node (PLN) sites (Figure 2), with the site of administration being the muscle. Parameter estimation was only conducted for the degradation of liposomes or antigen at each tissue site. The initial model estimates of the liposomes in the muscle were relatively good, with the exception of the fit at day one (PE = 32.7%), with a similar trend for the liposome in the PLN, with good fits to observed data except for day 14 (PE = 66.3%) (Table 2). The large variability in the 0.25-day observed sampling point and poorer fit at day one may suggest non-uniform tissue distribution following im injection. However, the lack of plasma data precluded further analysis of this to ascertain why there is this mismatch. In a similar fashion, the poor fit at days 14 in the PLN would suggest an alternative competing process resulting in the slower penetration into the tissue (Figure 5A). Yet, given the limited data points, this first approach at modelling the kinetics of liposome distribution from muscle into PLN is relatively successful. Indeed, the dose reaching the blood was significantly lower (<1 × 10^−6^% of the administered dose) and highlights the expected depot effect the muscle provides in maintaining the formulation within the dosing tissue.

For the antigen, we were able to describe the muscle kinetics relatively well with a higher PE at days 14 (65.9%) (Figure 5A and Table 2). Our model prediction for the PLN was predominately within the same order as magnitude as the reported data but with a higher PE than the muscle compartment. Given these concerns, we conducted a local sensitivity analysis to better identify parameters that lead to sensitivity to the residence of drug within the muscle and PLN (Figure 6). Both k_deg,muscle_ and f_e_ resulted in sensitivity towards AUC_muscle_ (Figure 6A), with k_deg,muscle_ being the most sensitive to changes in AUC_muscle_. This suggests that f_e_ only plays a minor role in governing the residency of the formulation within the muscle. For example, an 8-fold log change in f_e_ at a fixed k_deg,muscle_ of 0.051 day^−1^ resulted in 2-fold change in AUC_muscle_ (Figure 6A), whereas a fixed f_e_ utilised within the model resulted in a 3-log change in AUC_muscle_ over the range of k_deg,muscle_ simulated.

In the PLN, both f_e_ and k_deg,pln_ were able to affect AUC, with changes in f_e_ again being more prominent in influencing AUC_PLN_ (Figure 6C,D). At a f_e_ = 1, AUC_PLN_ is less sensitive to changes in k_deg,pln_ (Figure 6C), presumably as a result of the unhindered formulation flux between compartments. However, when fixed at 1 × 10^−8^, k_deg,pln_ resulted in a 5-fold change in AUC_PLN_ and became more important in controlling AUC_PLN_.

The local analysis has revealed the need to further quantify the ‘degradation’ process (i.e., antigen processing pathways: update, degradation, complex formation and presentation) to improve the sensitivity of the model to changes (estimates) for k_deg,tissue_ and f_e_. A more mechanistic approach to describe these pathways may better capture the kinetics events associated with k_deg,tissue_. In particular, when considering a global sensitivity analysis (Figure 7), with respect to the impact of both k_deg,muscle_ (Figure 7A) and f_e_ (Figure 7B) on percent dose, our predictions are within the 50th and 95th percentile in the muscle, but there is more uncertainty in the PLN, where a significantly wider range for the 95th percentile exists. This, coupled with the relatively low recovery, may require further studies to better characterise the reasons for such low accumulation (if it cannot be attributed to the size and impact on f_e_).

When extrapolating our model to humans, we have obtained first estimates of the potential injection site concentration (Figure 8) with a predicted mean C_max_ of 0.25 µg/mL and long half-life of 13.2 days in the deltoid muscle. This half-life is shorter than that predicted in the mouse quadriceps. The degradation of the antigen appears to have a more prominent role in governing its residency within the muscle (Figure 8A) rather than its ability to be cleared from the muscle tissue (Figure 8B). This may reflect the size of the liposomes (approximately 500 nm and highly cationic in nature) and their ability to be cleared from the depot site. Our approach to utilise percent-recovery data is a suitable first approximation at determining a static true ‘in-vivo’ parameter, particularly as the size of the nanoparticle would be expected to significantly hinder its drainage from the deport into the PLN and hence, our first-principals approach is justified.

In summary, this study has demonstrated the potential to apply the principles of pharmacokinetics to assess in-vivo data from liposome studies, but also shows how we can apply pharmacokinetics in a mechanistic approach to allow cross-species extrapolation. It must be noted that caveats exist and should be considered when the reader wishes to apply pharmacokinetics principles to data sets.

Lack of plasma data: Key to any pharmacokinetics study is the requirement to sample from the blood/plasma. By sampling from the plasma (in addition to the target site), the reader is then able to take account of distributional processes to other tissues and hence move the model towards a more mechanistic full-PBPK model rather than the semi-mechanistic PBPK model presented in this study.

Interpretation of tissue partition: The passage of a therapeutic entity through a cell requires partitioning across cellular membranes. This is often accounted for by the tissue partition coefficient (kp) [33,34], which can readily be predicted from the physiochemical properties of low molecular weight compounds. Our approach did not address this issue and assumed there was no inherent permeability barrier, with transvascular flux being the main factor governing the entry/exit to a tissue. Possessing prior knowledge of the formulation system, we surmised that the liposomes would be unable to partition out due to their size (~500 nm) and hence have focused on the depot effect provided by the muscle. For other nanoparticles, this may not be the case, and kps would need to be determined. By possessing plasma data along with some additional tissue data, it would be possible to obtain first estimates of the kp for each tissue of interest.

Modelling antigen pharmacokinetics: Our approach to the pharmacokinetic modelling of the antigen element of the formulation is simplistic when considering the multitude of processes involved in the absorption, distribution, metabolism and elimination (ADME) processing of the antigen, along with non-linear processes such as FcRn-mediated transcytosis and target site-mediated processes. A driving force for the selection of an appropriate model is the availability of data that support model development, and in this case we have presented a first-principles approach to capture some elements of the antigen pharmacokinetics.

## 5. Conclusions

In conclusion, this study showed an approach for predicting the in-vivo fate of DDA:TDB:H1 in humans and demonstrated the role that formulation degradation and fraction escaping the depot site can play in the overall depot effect within the site of administration.

## Figures and Tables

**Table 1:** Anatomic and physiological parameters used in the physiologically based pharmacokinetics (PBPK) models; PLN: popliteal lymph node ^1^.

Compartment	Flow (L/Day) Symbol	Mouse	Human	Volume (L) Symbol	Mouse	Human
Plasma	Q_plasma_	-	-	V_plasma_	9.44 × 10^−4^ [24]	3.13
Quadriceps	Q_muscle_	2.60 [24]	32.04 [25]	V_muscle_	1.6 × 10^−4^ [26]	0.19 [25]
PLN	Q_PLN_	2 × 10^−3^ [27]	0.52 [27]	V_PLN_	5 × 10^−6^ [26]	3.5 × 10^−3^ [27]
Rest of Body	Q_rest_	18.10	3.02 × 10^4^	V_rest_	2.6 × 10^−2^	67.68

^1^ Except where indicated, all parameters were taken from [23].

**Table 2:** Summary of model-fitted pharmacokinetic parameters.

**Liposome**	
K10	0.0306 day^−1^
t_1/2_	22.6 days
MRT	32.6 days
AIC	49.54
**Antigen**	
k10	0.34 day^−1^
k12	22.26 day^−1^
k21	77.58 day^−1^
t_1/2α_	0.0069 day
t_1/2β_	2.62 days
MRT	3.78 days
AIC	37.4

Parameters best fit a 1-compartment model for the liposome and a 2-compartment model for the antigen. MRT: mean residence time; t_1/2α_: distribution half-life; t_1/2β_: terminal half-life; AIC: Akaike information criterion.

**Table 3:** Precision error calculations.

Time (Days)	% Precision Error			
	Muscle		PLN	
	Liposome	Antigen	Liposome	Antigen
0.25	16.4	13.5	-	-
1	32.7	5.3	28.4	32.7
4	3.9	13.8	13.90	41.5
14	3.8	65.9	66.3	74.0

**Table 4:** Parameter estimates for k_deg_.

Compartment	Degradation Constant (Day^−1^ ± SD)		
	Symbol	Liposome	Antigen
Plasma	-	-	-
Quadriceps	k_deg,muscle_	0.051 ± 0.008	0.320 ± 0.028
PLN	k_deg,pln_	0.132 ± 0.004	0.280 ± 0.013
Rest of Body	k_deg,rest_	0.091 ± 0.040	0.110 ± 0.030
